# Supplementary material for: Temporal and spatial composition of the tumor microenvironment predicts response to immune checkpoint inhibition
Source: bioRxiv. 2025 Jan 28:2025.01.26.634557. Preprint. [Version 1] doi: 10.1101/2025.01.26.634557 (PMC11838242; doi:10.1101/2025.01.26.634557)
Supplement: Supplement 1 [file media-1.pdf]

Extended Data Figure 1. ROI selection and sample summary

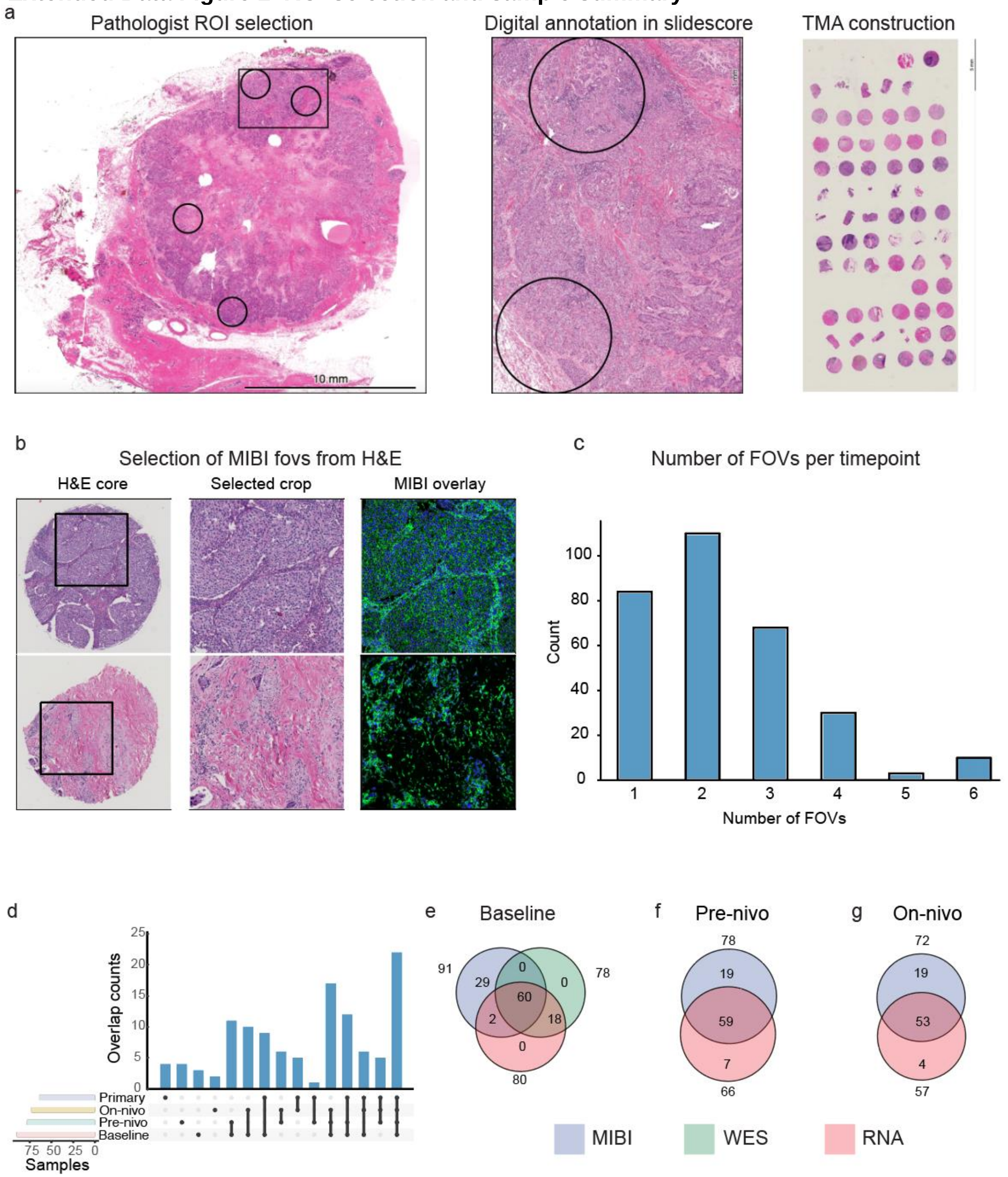

a) Workflow for selecting cores to be included in the study. Hematoxylin and eosin (H&E) slides were digitized in slidescore.com, annotated by a dedicated breast cancer pathologist, and demarcated with areas of interest. Each identified region was punched with a 1.5mm core and placed onto a tissue microarray (TMA). **b**) Illustrative examples of the H&E image of the entire core, the cropped inset of the portion of the core selected for MIBI analysis, and the corresponding MIBI image generated from the selected region. **c**) Histogram showing the number of fields-of-view (x-axis) acquired on MIBI per tumor sample (y-axis). **d**) Upset plot showing the overlap of distinct timepoints of MIBI data across the patients in the cohort. **e**) Venn diagram showing the overlap between DNA, RNA, and MIBI data for baseline samples. **f**) Venn diagram showing the overlap between RNA and MIBI data for pre-nivo samples. **g**) Same as f) for on-nivo

Extended Data Figure 2. Antibody panel validation

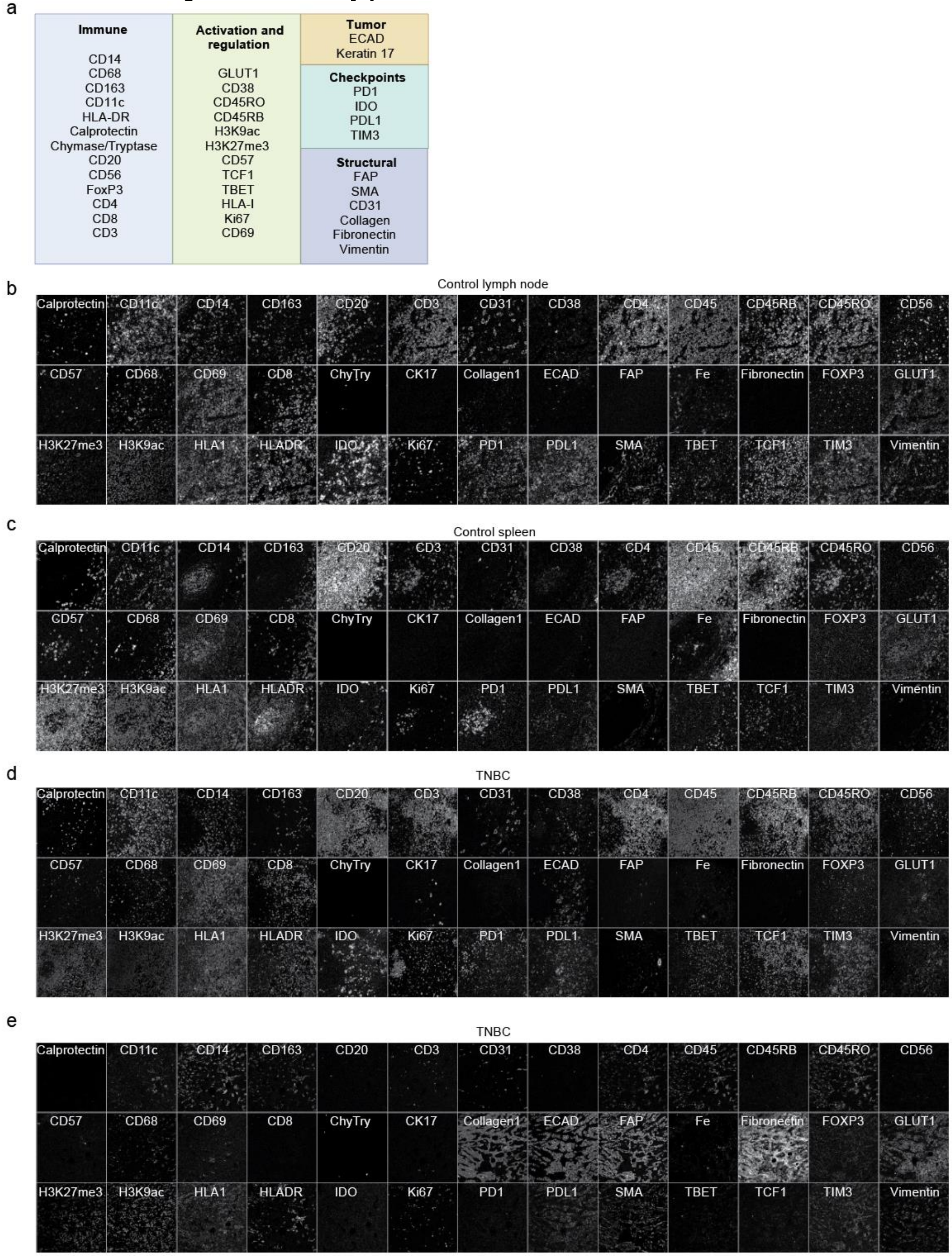

a) MIBI panel, including immune markers, tumor markers, checkpoint markers, structural markers and markers related to activation and regulation. **b**) Single-plex images of each marker in the panel in a control lymph node sample. **c**) Same as b) for a control spleen sample. **d**) Same as b) for first representative TNBC sample. **e**) Same as b) for second representative TNBC sample.

Extended Data Figure 3. Rosetta image compensation

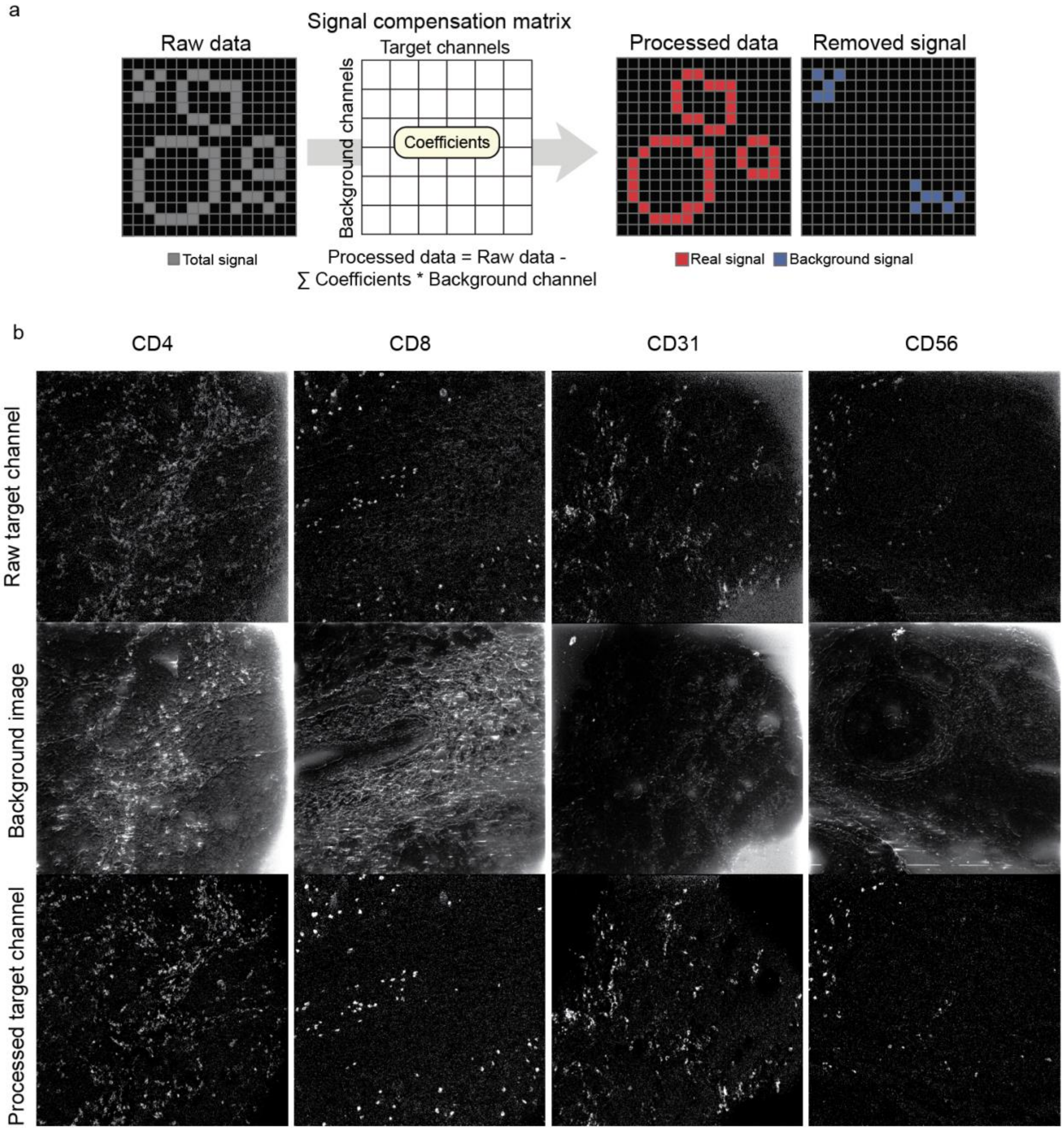

a) Cartoon illustrating the Rosetta image compensation process, where background signal is subtracted using a compensation matrix to produce cleaned up images. **b**) Representative examples of different channels before compensation (top), after compensation (bottom), along with the corresponding background channel used for compensation (middle) from the same image.

Extended Data Figure 4. Image normalization and quality control

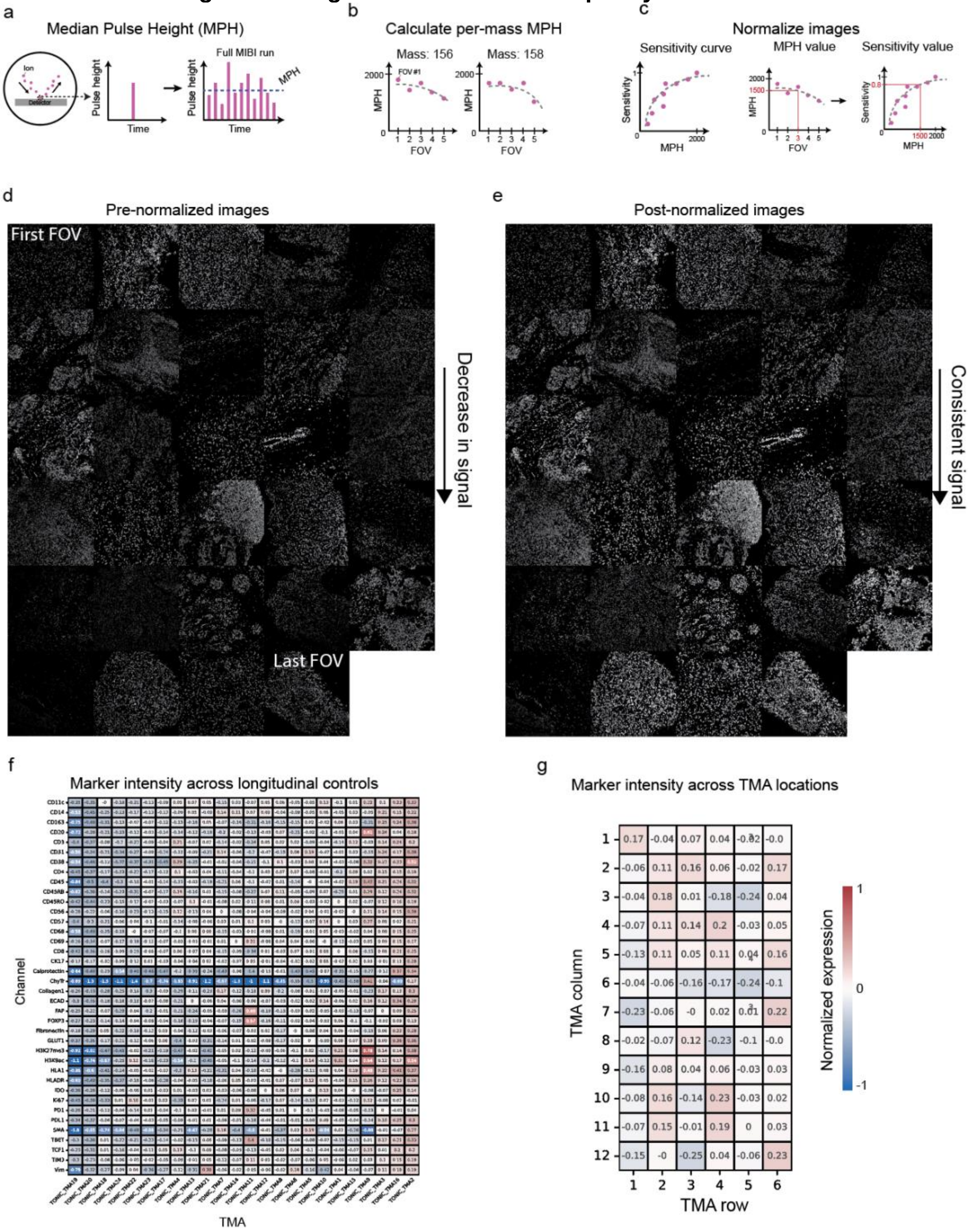

a) Cartoon illustrating how median pulse height (MPH) is calculated. **b**) Cartoon showing how a per-mass MPH curve is fit for an imaging run. **c**) Cartoon showing how the sensitivity curve is combined with the per-mass MPH curve to correct for changes in sensitivity over the course of an imaging run. **d**) Representative stitched image showing the uncorrected images from a single channel across an entire run, stitched in acquisition order from the start of the run (top left) to the end of the run (bottom right). **e**) Representative stitched image showing the corrected intensities from the images in d) following MPH normalization. **f**) QC plot showing the normalized expression in the control tissues of all the markers in the panel (y-axis) across the different TMAs in the cohort (x-axis). **g**) QC plot showing the normalized expression per field-of-view, plotted with the same row/column location as in the actual TMA.

Extended Data Figure 5. Cell segmentation

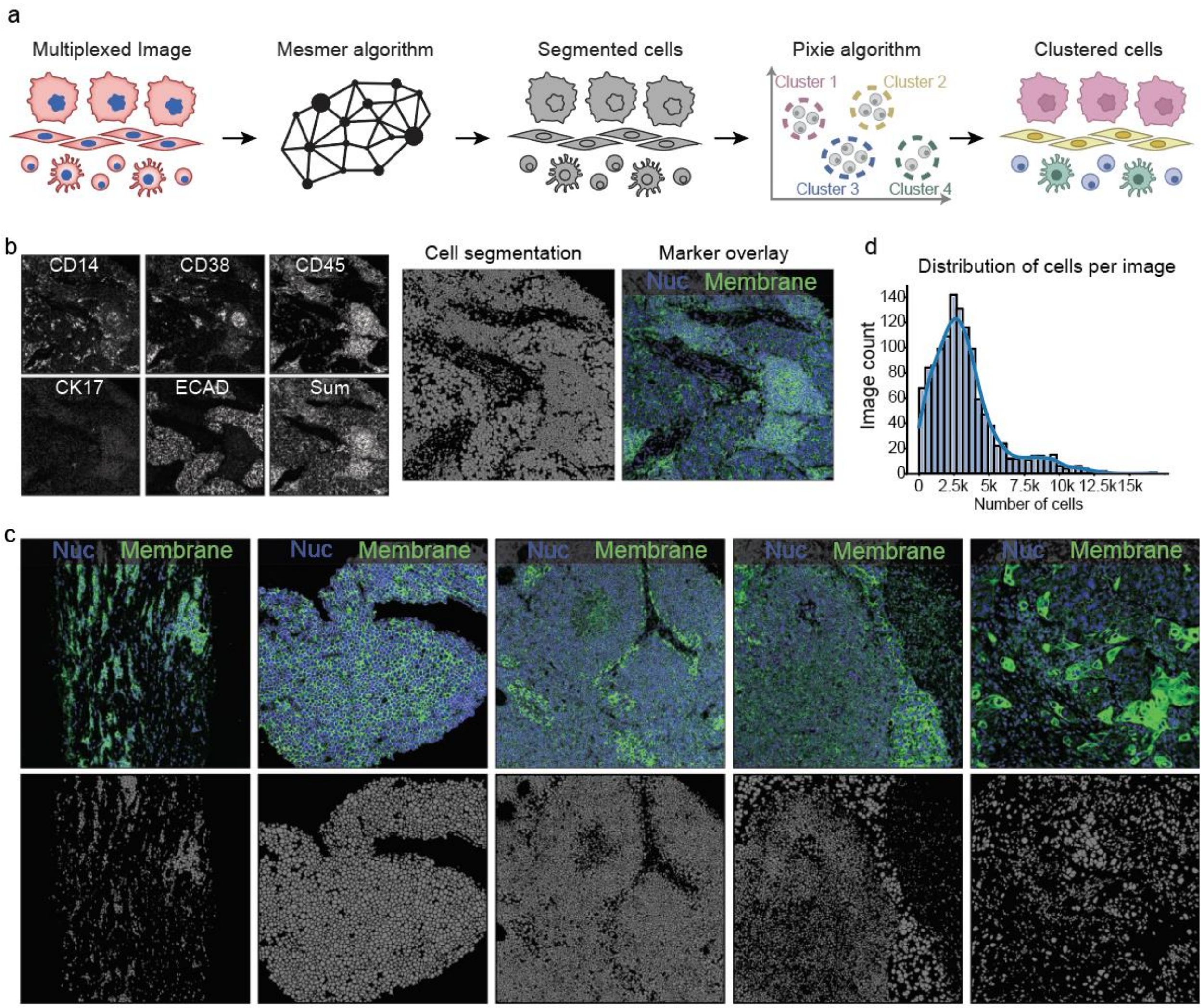

a) Cartoon of the segmentation and clustering pipeline. **b**) Representative image showing the different membrane markers that were combined together into a single membrane channel for segmentation (left), the resulting segmentation mask generated by Mesmer (middle), along with an overlay of segmentation mask and channels used for segmentation (right). **c**) Additional representative images of showing the segmentation channels overlaid with the segmentation outlines (top), along with the segmentation mask on its own (bottom). **d**) Histogram showing the number of cells per image across all images in the cohort.

Extended Data Figure 6. Cell clustering and abundance

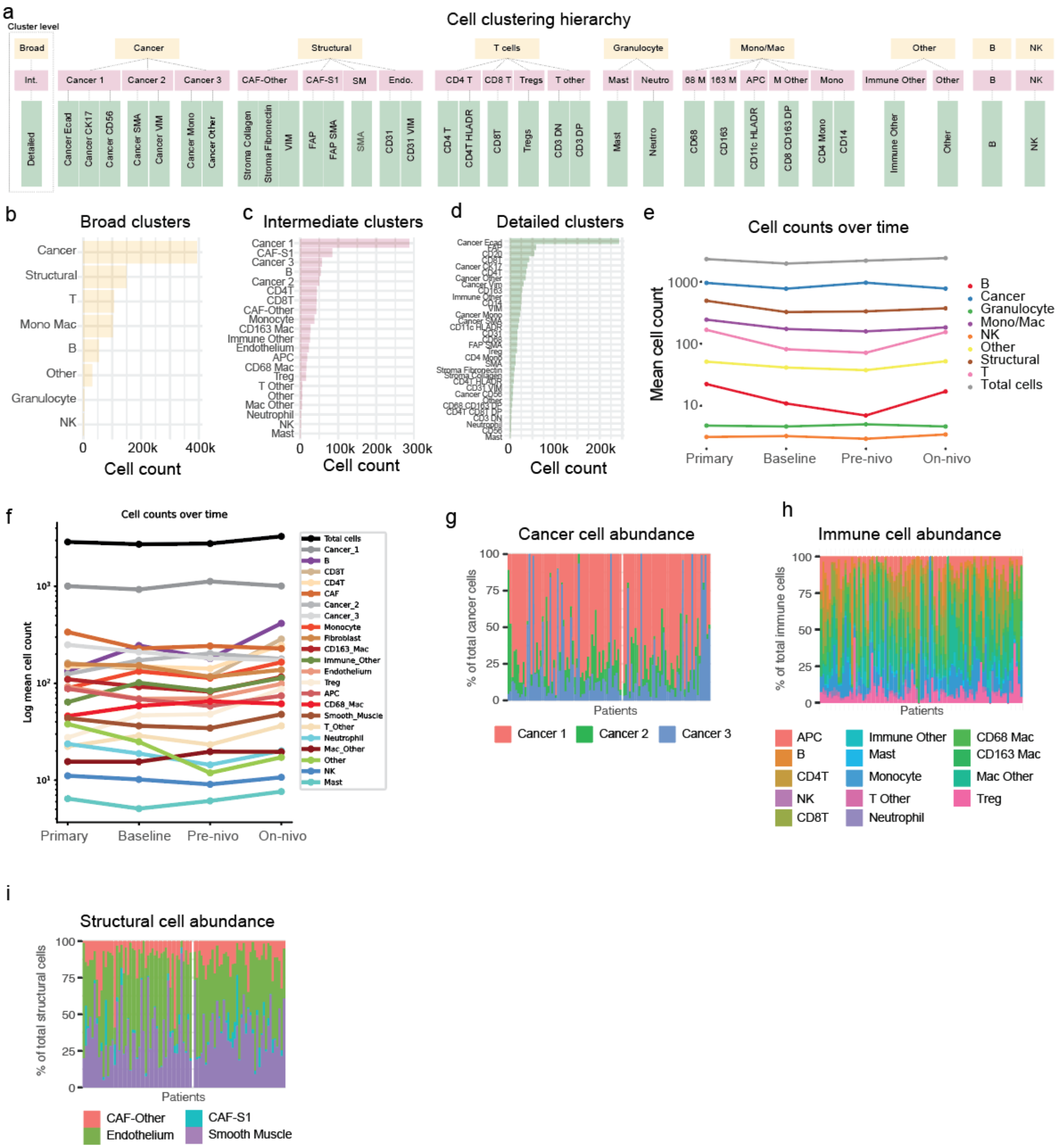

a) Diagram illustrating the three levels of cell clustering (broad, intermediate, detailed), and the relationship between clusters in each level. **b**) Number of cells of each cell type in the broad cluster. **c**) Number of cells of each cell type in the intermediate clusters. **d**) Number of cells of each cell type in the detailed clusterings. b, c, d are all based on all primary and metastatic tumors in the sample set. **e**) Mean count of cell types (broad clusters) for each timepoint. **f**) Mean count of cell types (intermediate clusters) for each timepoint. **g**) Average proportion of each intermediate cancer subpopulation in metastatic samples. **h**) Average proportion of each intermediate immune subpopulation in metastatic samples. **i**) Average proportion of each intermediate structural subpopulation in metastatic samples

Extended Data Figure 7. Feature extraction pipeline cartoons

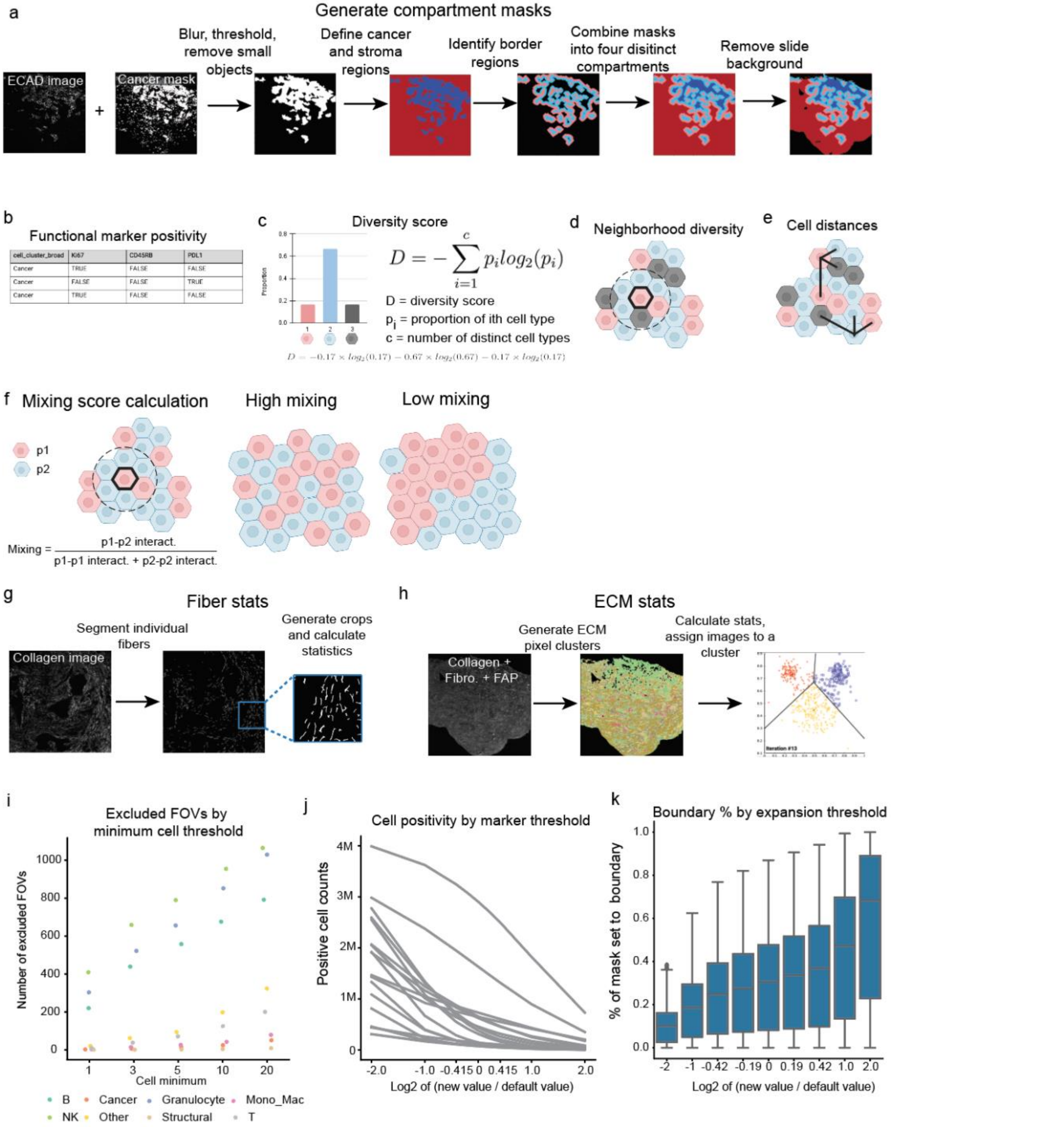

a) Cartoon illustrating how compartment masks are defined. **b**) Table illustrating how functional marker thresholds are used to binarize cells into positive or negative for each marker; these assignments are then used to generate positivity proportion statistics per image. **c**) Schematic showcasing how diversity scores are calculated. **d**) Cartoon showing how the radius around a cell is used for neighborhood diversity. **e**) Cartoon showing how cell distances are used to compute the linear distance feature. **f**) Cartoon showing how the mixing score is calculated, along with examples of high and low mixing. **g**) Schematic showing how the fiber segmentation pipeline is used to generate features. **h**) Schematic showing how the extracellular matrix pipeline is used to generate features. **i**) Number of images removed from analysis as a function of different minimum cell thresholds, stratified by cell type. **j**) The number of functional marker positive cells across different thresholds. **k**) The proportion of the image assigned to the border compartment across different expansion thresholds.

Extended Data Figure 8. Quantification of features across tumor compartments

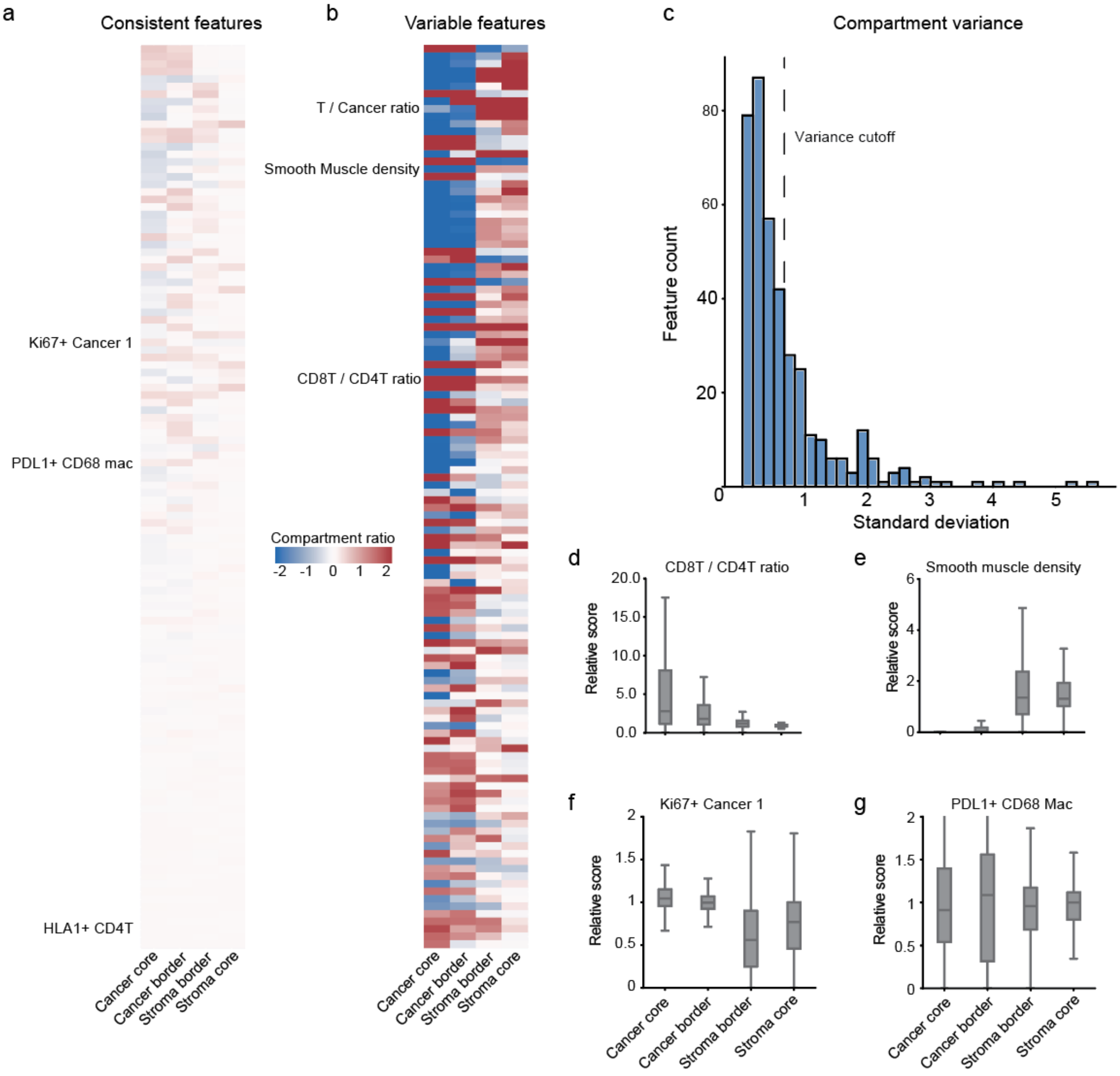

a) Heatmap showing features that are consistent across compartments. Each row represents a different feature, and each column is a distinct compartment. Features are normalized to the whole image value. **b**) Same as a), but for features that are enriched in specific compartment(s). **c**) Histogram showing threshold used to identify varying vs. non-varying compartment features. **d**) Distribution of the CD8 T / CD4 T ratio, a feature that changed across compartments. **e**) Distribution of Smooth Muscle density, a feature that changed across compartments. **f**) Distribution of Ki67 positivity in Cancer 1, a feature that did not change across compartments. **g**) Distribution of PD-L1 positivity in CD68 macrophages, a feature that did not change across compartments.

Extended Data Figure 9. Univariate outcomes pipeline robustness

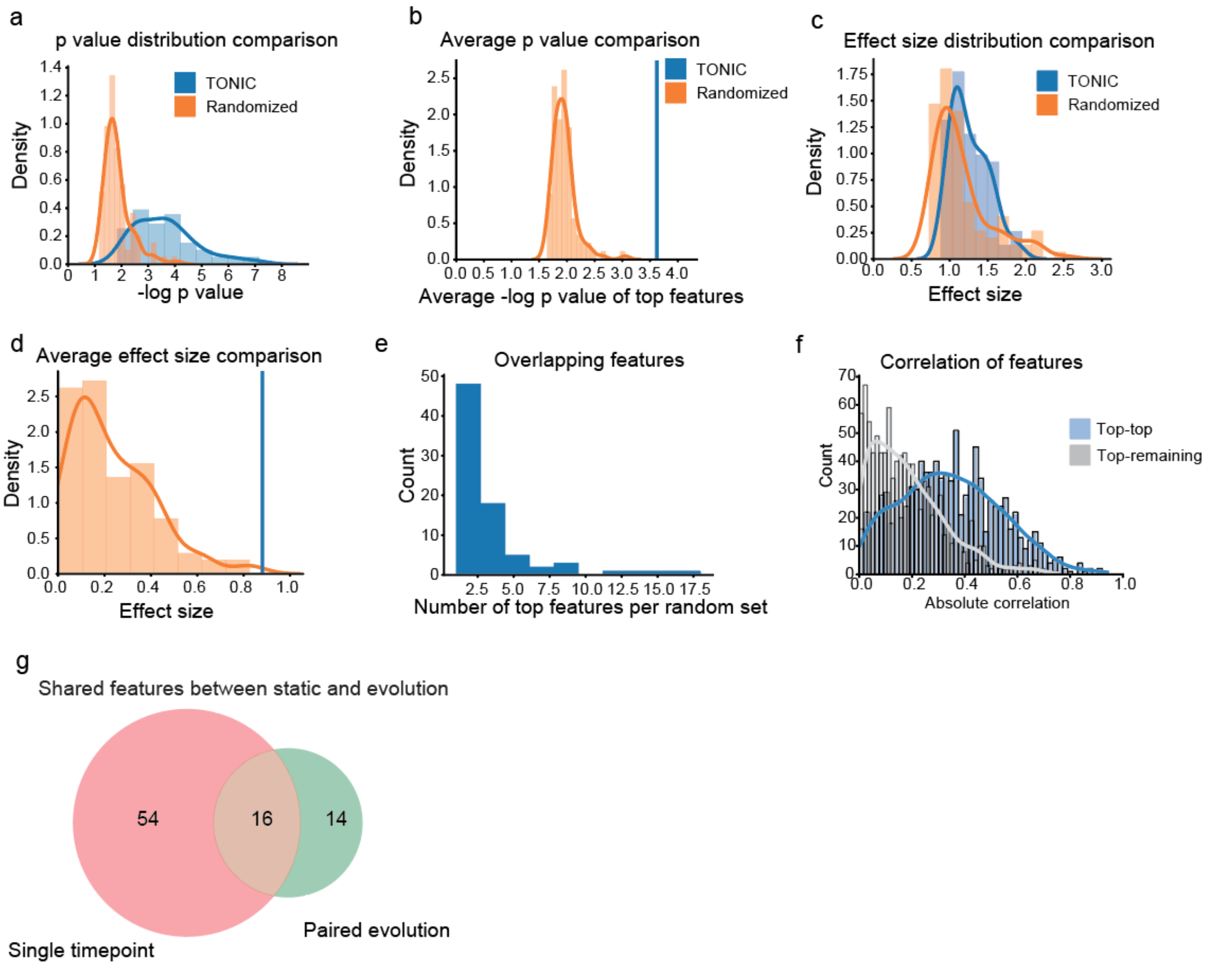

a) Distribution of p-values from the top features 100, as well as distribution of p-values from the top 100 features recalculated after the labels have been randomized. **b**) Average p-value in the top features, as well as distribution of average p-values across 100 different randomizations. **c**) Distribution of effect sizes from the top 100 features, as well as distribution of effect sizes from top 100 features recalculated after the labels have been randomized. **d**) Average effect size in the top features, as well as distribution of average effect sizes across 100 different randomizations. **e**) Number of times the same feature come up in different randomizations. **f**) Correlation between top features, compared with correlation between top features and other features. **g**) Overlap in features identified when examining each timepoint independently, compared to looking at the change in each feature across timepoints.

Extended Data Figure 10. Outcomes associations

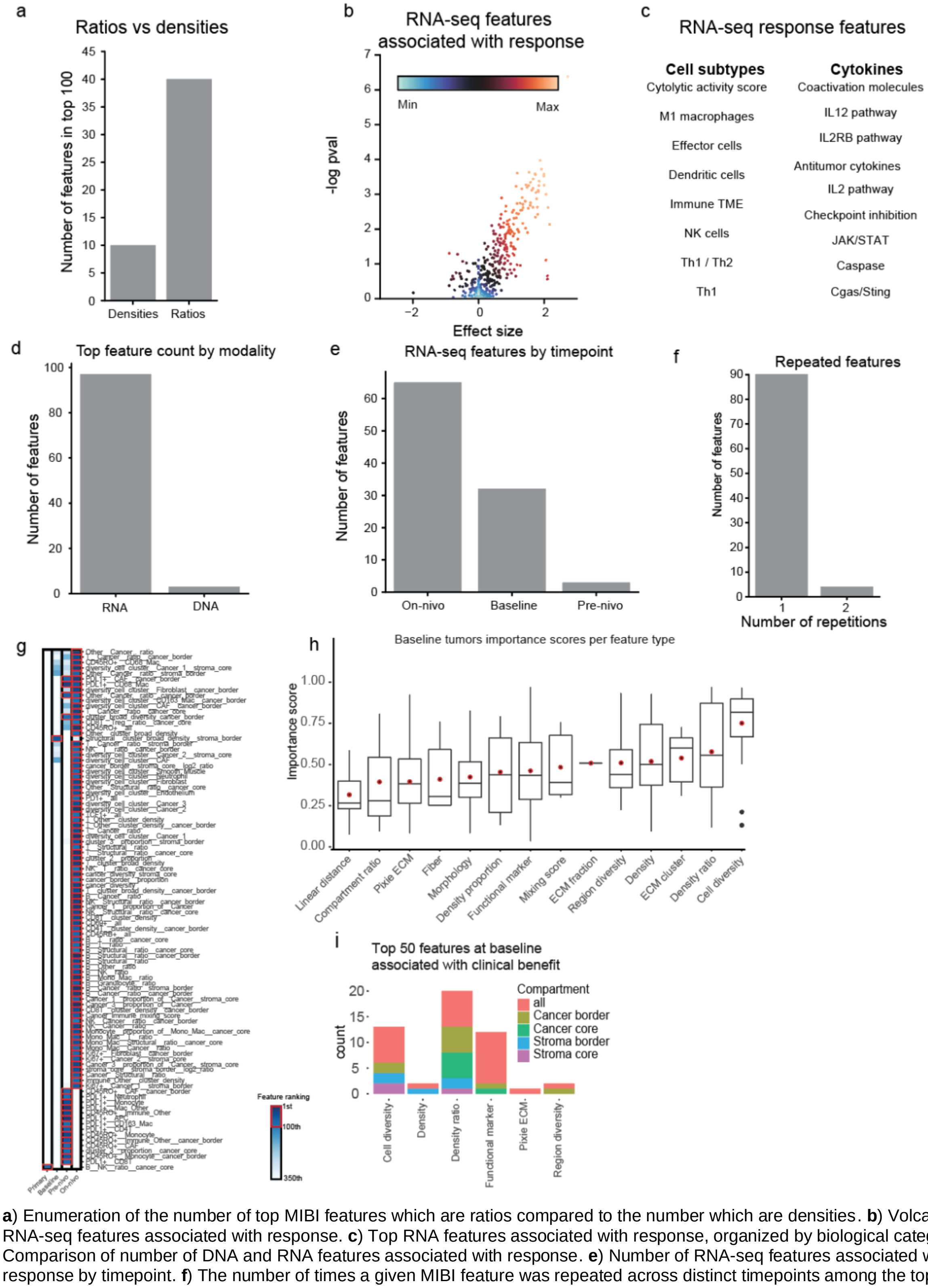

a) Enumeration of the number of top MIBI features which are ratios compared to the number which are densities. **b**) Volcano plot of RNA-seq features associated with response. **c**) Top RNA features associated with response, organized by biological category. **d**) Comparison of number of DNA and RNA features associated with response. **e**) Number of RNA-seq features associated with response by timepoint. **f**) The number of times a given MIBI feature was repeated across distinct timepoints among the top 100 features. **g**) Same as Fig. 4b, but with all labels included. **h**) Types of features associated with response from baseline metastatic timepoint. **i**) Top 50 features associated with response from baseline timepoint, colored by compartment and sorted by feature type.

Extended Data Figure 11. Timepoint-specific features

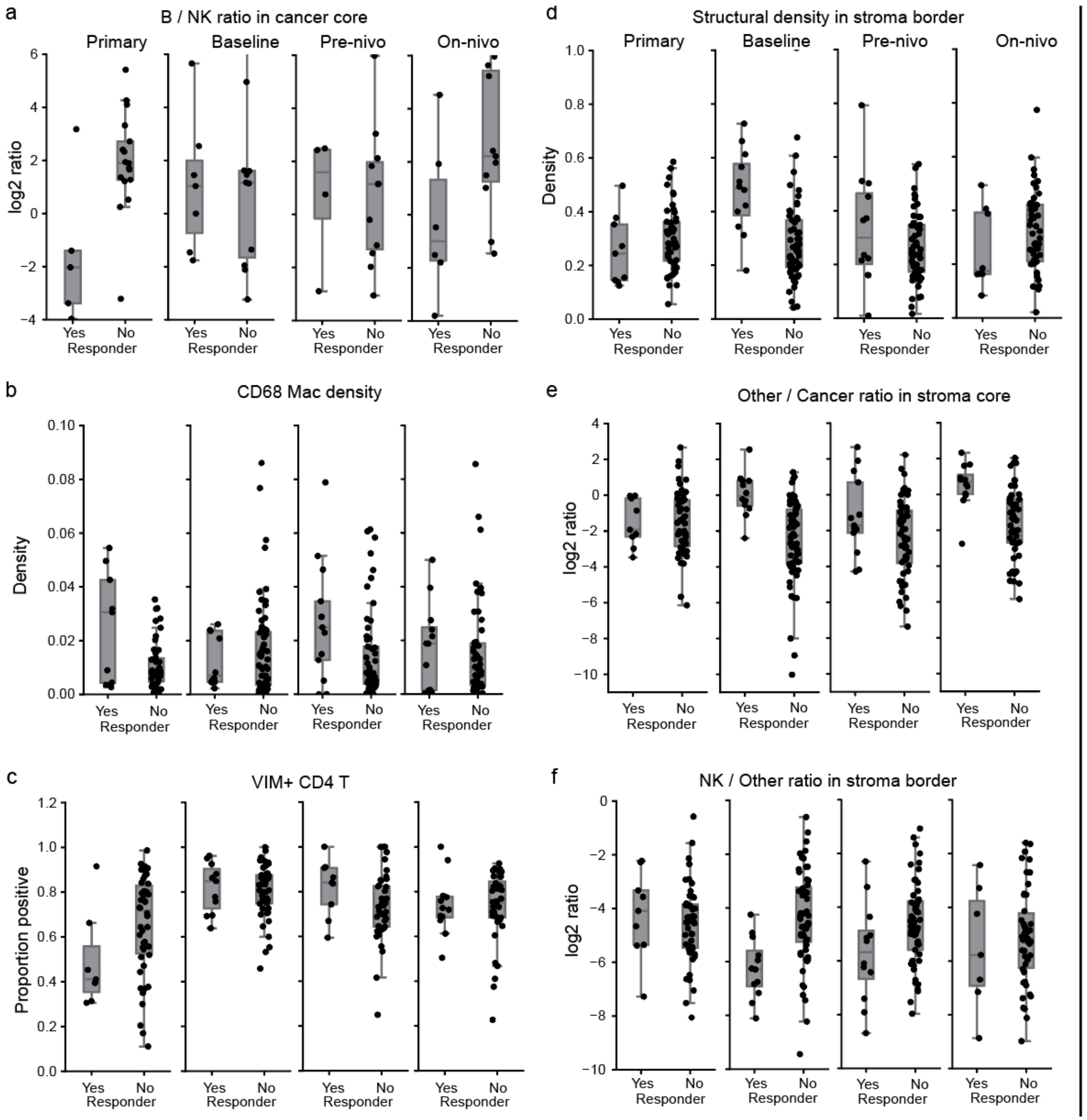

a) Box plots showing the distribution of the B cell / NK cell ratio feature in responders and non-responders, across the four distinct timepoints in the cohort. **b**) Same as a), with CD68 macrophage density as the feature. **c**) Same as a), with Vimentin positive CD4 T cells as the feature. **d**) Same as a), with structural cell density in the stroma border as the feature. **e**) Same as a), with the ratio of other to cancer cells as the feature. **f**) Same as a), with the ratio of NK cells to other cells as the feature.

Extended Data Figure 12. Multivariate modeling

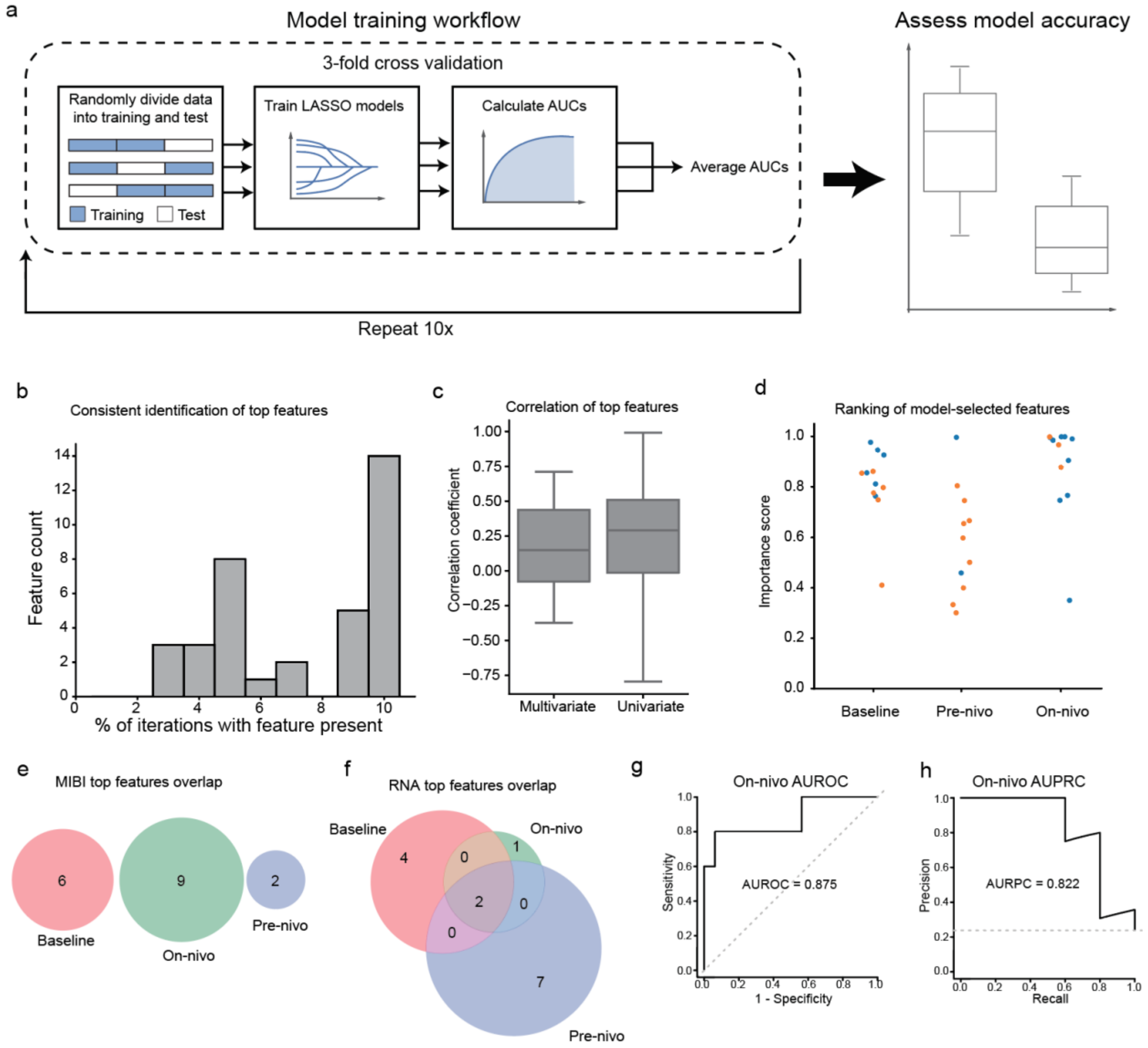

a) Diagram illustrating the cross validation approach used for model training. **b**) Histogram showing the number of times the top features were selected across the 10 different iterations of model training. **c**) Comparison between the correlation of the top features identified by the model and the top features from the univariate analysis. **d**) Comparison of the importance score of the top features identified by the model across distinct timepoints, stratified by MIBI vs. RNA. **e**) Overlap across timepoints of top features identified by the MIBI models. **f**) Overlap across timepoints of top features identified by the RNA models. **g**) AUROC evaluated for the on-nivo MIBI data using separate train, val, test split without cross validation. **h**) Same as above, for AUPRC.
